# Electrically Conductive and Antimicrobial Agro-Food Waste Biochar Functionalized with Zinc Oxide Particles

**DOI:** 10.3390/ijms23148022

**Published:** 2022-07-21

**Authors:** Zélia Alves, Nuno M. Ferreira, Gonçalo Figueiredo, Sónia Mendo, Cláudia Nunes, Paula Ferreira

**Affiliations:** 1 Department of Materials and Ceramic Engineering, CICECO—Aveiro Institute of Materials, University of Aveiro, 3810-193 Aveiro, Portugal; zeliaralves@ua.pt; 2 Department of Chemistry, CICECO—Aveiro Institute of Materials, University of Aveiro, 3810-193 Aveiro, Portugal; 3 Department of Physics, i3N, University of Aveiro, 3810-193 Aveiro, Portugal; nmferreira@ua.pt; 4 Department of Biology, CESAM, University of Aveiro, 3810-193 Aveiro, Portugal; gfigueiredo@ua.pt (G.F.); smendo@ua.pt (S.M.)

**Keywords:** kidney-bean pods, pyrolysis, solvothermal synthesis, carbon material, circular economy

## Abstract

Carbonaceous materials derived from biomass have been used as sustainable platforms for the growth of ZnO particles aiming the production of functional composite fillers. Kidney-bean pods were pyrolyzed by applying an experimental design that demonstrates that the specific surface area (S_BET_) of biochar is improved with increasing pyrolysis temperature combined with a short air-oxidation time. Meanwhile, the graphitization degree and the electrical conductivity (EC) of biochars were negatively affected by increasing the air-oxidation time. The biochar sample with the higher EC and the one with the higher S_BET_ were selected to be functionalized with ZnO particles by a solvothermal methodology, obtaining composites with an EC and S_BET_ properties superior to the ZnO-rGO composite, in addition to a similar antibacterial activity. The developed ZnO-biochar composite structures, which are more ecological and biocompatible than the ZnO composites derived from graphene sheets, can be applied as electrically conductive and active fillers.

## 1. Introduction

Carbon-based fillers such as carbon black, graphene derivatives, and carbon nanotubes have been extensively and efficiently explored owing to their high electrical conductivity (EC), large specific surface area, and high amenabilities to surface functionalization [1,2,3,4]. Although these carbon materials exhibit unique properties, they are produced from feedstocks derived from petroleum involving relatively time-consuming, non-sustainable, and expensive methodologies [5]. Focusing on graphene derivatives, large chemical inputs are required to produce graphene oxide (GO) from graphite when prepared via the commonly known Hummers’ method, which is not economically affordable and environmentally sustainable [6]. In addition, even following green methodologies using hydro- or solvothermal systems, chemicals are needed to restore the conjugated structure and reduce the GO, forming reduced graphene oxide (rGO) with enhanced EC values [7]. Hence, there is a great need to search for new materials derived from natural abundant resources and green synthesis to produce functional graphene-like carbon structures, meeting the growing applications of such materials [8].

Recent research has used a variety of biomass sources as a carbon-rich structure to be pyrolyzed in the absence of oxygen at different heating rates [9,10,11]. During the thermal decomposition of the biomass, a solid carbon-rich material, called biochar, is obtained; it is composed of amorphous carbon with a certain graphitic order and abundant surface functional groups [12]. Agro-food wastes used as biomass to produce carbon-based materials are a good alternative for the graphite replacement because they are raw materials that are comparatively cheaper, renewable, and abundant. For example, the worldwide production of dry beans in 2017 exceeded 31.4 million metric tons, leading to the generation of a high quantity of agro-food wastes (e.g., dried stems or pods) [13]. There is a growing demand to find new sustainable routes to valorize these wastes, which usually requires management and disposal, beyond use in animal feed or burning for heat production.

The type of biomass and the pyrolysis conditions, namely the pyrolysis temperature, the residence time, and the heat transfer rate, significantly influence the physical and chemical properties of as-prepared biochar [14]. Pyrolysis temperatures beyond 700 °C and longer residence times are needed to transform the disordered carbon biomass into an ordered graphitic structure with a higher surface area and EC [15,16]. In addition, the surface chemistry of biochars can be changed after pyrolysis by thermal air oxidation, a methodology considered to increase the pore and surface area but also to increase surface-oxygen functionality with hydroxyl, carboxyl, and carbonyl groups [17,18]. Contrary to the conventional oxidation methodologies using aggressive reagents (e.g., strong acids, ozone, and hydrogen peroxide) [19], biochar oxidation by air exposure is a solvent-free methodology and can be easily applied during the cooling process after biochar production [18]. The oxygenated groups in the surface not only affect the physicochemical properties of biochar but also facilitate the surface modification with other materials, including metal oxides, combining functionalities in one composite material [20]. Among all the metal oxides, ZnO is a desirable antimicrobial, antioxidant and UV-light barrier that has low toxicity, is biocompatible, and is cost-effective, which are characteristics that make it applicable in the environmental and food sector [21,22]. Until now, the hybridization of carbon-based materials, such as rGO or even biochar, with ZnO nanoparticles has produced high-performance composites to be employed in contaminant remediation by adsorption of heavy metals [23,24] or in the photocatalytic activity with organic compounds and dyes [25]. However, there is limited knowledge about the performance of ZnO-biochar composites on the electrical and antimicrobial activities, which are important features to be imparted to a biopolymeric matrix through the addition of this composite as a filler. In the food area industry, an electrically conductive biopolymeric composite material can be applied as a food packaging to inactivate the growth of microorganisms of packaged food products using electric pulses [26]. Additionally, the antimicrobial properties of the composite can also prevent microbial growth on the food surface by direct contact of the packaging material with the food.

The novelty of this work consists of using for the first time a biochar derived from kidney-bean pods (KBP) as eco-friendly carbon support to produce electrically conductive and antimicrobial ZnO composites with great potential as active fillers in the food-packaging industry. To fulfill this, an inexpensive and single-step strategy following the Taguchi experimental design was used to produce a variety of biochar structures through KBP pyrolysis, without the use of any external chemical agents and applying the circular economy principles. The influence of pyrolysis temperature and time as well as air-oxidation time as post-pyrolysis treatment on electrical conductivity and specific surface area of biochar were investigated using the experimental design to reduce the number of experiments, saving research time. The best-performance biochar samples concerning the Brunauer, Emmett, and Teller (BET) specific surface area (S_BET_) and EC were in a second approach functionalized with zinc-oxide particles by a simple and cost-effective solvothermal procedure, as an alternative to the rGO platform commonly used. A biochar structure with a higher surface area supports the growth and the stability of metal-oxide particles and prevents them from agglomeration. In addition, the effectiveness of antibacterial-activity materials, including ZnO composites, has been reported to take advantage of a large surface area [27]. On the other hand, the design of a composite with high electrical conductivity is a key factor to give conductivity to biomaterials for use in food sterilization. The characterization of synthesized ZnO-biochar (ZnO-C) composites, in terms of structure, morphology, S_BET_, electrical conductivity, and antimicrobial properties, was compared with the ZnO and ZnO-rGO as benchmark composite. Thus, the potential of biochar as sustainable carbon support for the replacement of graphene carbon-based materials in ZnO composites will be evaluated.

## 2. Results and Discussion

**Figure 1:**
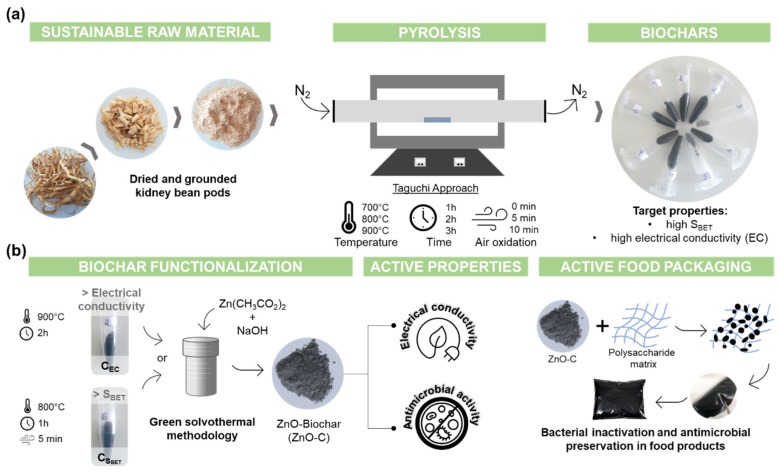
Workflow of (**a**) biochar production derived from kidney-bean-pod pyrolysis using a Taguchi approach to obtain biochars with distinct characteristics, and (**b**) biochar functionalization with ZnO particles by a solvothermal methodology obtaining composites to be used as fillers in a biopolymeric matrix for active food packaging.

Biochars derived from dried kidney-bean pods were obtained by pyrolysis (Figure 1a). Under visual analysis, the biochar samples resultant from run 3 (900C_1h_10air) and run 5 (800C_2h_10air) presented a greyish color compared to all other samples, which showed a blackish color, demonstrating the effect of the pyrolysis parameters in the biochar characteristics. The influence of the selected factors (T, time, and air) on the S_BET_, I_D_/I_G_ ratio and EC were analyzed according to the Taguchi method by the calculated S/N ratio and analysis of variance (ANOVA). Two resultant biochars with distinct characteristics, namely high specific surface area (evaluated by S_BET_) and EC, were selected to be impregnated with ZnO particles using a green solvothermal method to obtain biological active composites for food packaging materials (Figure 1b). The efficacy of these ZnO-C composites in terms of the S_BET_, electrical, and antimicrobial activities was tested and compared with the ZnO-rGO composite and pristine ZnO particles.

### 2.1. Characterization of Biochars

**Figure 2:**
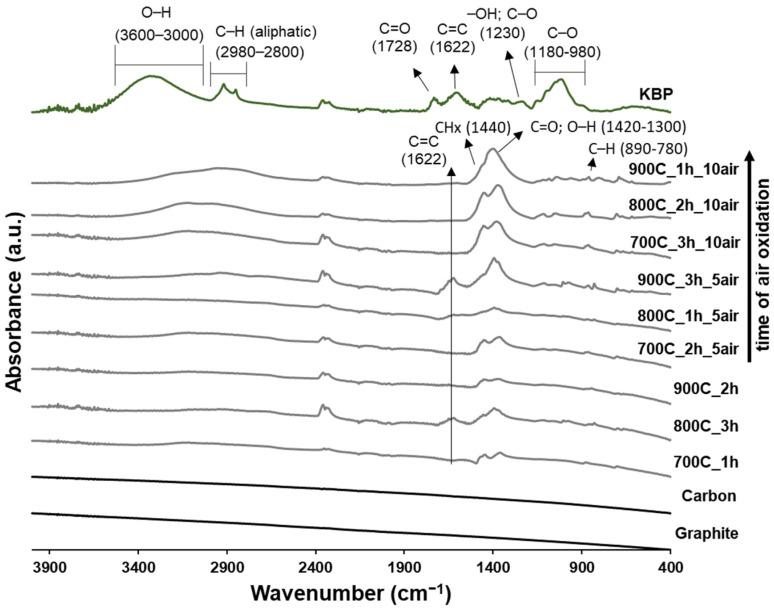
ATR–FTIR spectra of biomass (kidney-bean pods, KBPs) and the biochars resulting from pyrolysis using different time, temperature, and air oxidation conditions. The spectra of carbon and graphite are displayed as a reference.

The ATR–FTIR spectra of biomass raw material and the different biochars produced are shown in Figure 2. The spectrum of kidney-bean pods (KBPs) showed a broad band at 3600–3000 cm^−1^ which is attributed to –OH vibration stretch of intramolecular hydrogen bonds from the cellulose molecule. The adsorption in the region between 2980 and 2800 cm^−1^ is assigned to the –CH_x_ asymmetric stretching vibration of aliphatic hydrocarbon groups. The band at 1728 cm^−1^ is associated to C=O from conjugated aromatic carbonyl/carboxyl groups representative mostly of hemicellulose and the band at 1622 cm^−1^ is attributed to the C=C vibration of aromatic groups that are present in lignin [28]. The band at 1230 cm^−1^ represents the peak associated with the –OH groups of phenolic compounds or C–O stretching vibration (e.g., acetyl esters). The strong band at 1180–980 cm^−1^ is ascribed to the C–O stretching associated with oxygenated functional groups (aromatic ring C–O stretching) of carbohydrates, namely cellulose, hemicellulose, and lignin [29]. Substantial chemical transformations are observed when the KBP is pyrolyzed in all the tested conditions, particularly with the decrease in the intensity of the bands related with –OH (3600–3000 cm^−1^), aliphatic–CH_x_ (2980–2800 cm^−1^), C=O (1728 cm^−1^), and C–O (1180–980 cm^−1^) groups. This could be explained by the instability and weakness of these linkages during the degradation and dehydration of cellulosic and ligneous components [30]. Even after the pyrolysis treatment, biochar spectra highlighted peaks associated with the aliphatic chains (1440 cm^−1^, CH_3_ and CH_2_ groups [31]), but also evidenced a band around 1425–1300 cm^−1^ ascribed to C=O and/or –OH deformation vibrations [32], indicating the presence of some residual oxygen-containing functional groups. Despite this, the spectra of biochar samples without the air-oxidative treatment closely resemble the FTIR spectra displayed by pure graphite and carbon, demonstrating a limited presence of functional groups on its surface. When the oxidative treatment was applied on biochar samples, it was observed that increasing the time of air exposition the intensity of bands located between 1500–1200 cm^−1^ increased but seemed to also be dependent on time and temperature applied during the thermal decomposition of biomass. Thus, the air oxidation after the biomass pyrolysis is effective in introducing defects to the biochar carbon structure, notably evidencing the introduction of O-containing functional groups and aliphatic groups. The intensity of this last group increases probably due to the opening of aromatic condensed rings, a similar behavior already reported when biochar was treated with H_2_O_2_ [33].

The crystal-structure properties of biochars were determined with X-ray diffraction analysis. The XRD patterns (Figure S1) of biochars are complex because they showed several sharp and narrow peaks, indicating the presence of various inorganic components. The peaks observed showed the samples crystallinity and can be attributed to oxidative minerals based on K, Ca, Mg, and Si, as confirmed by EDS (Figure S2). However, exhaustive peak identification and assignment become complicated due to the peaks overlapping. By comparing the biochar diffractograms, it is visible that inorganic crystalline planes and their intensities differ between the samples, which suggests that temperature, residence time, and air-oxidation time are influent factors on biochar crystallinity. Biochar samples produced without the air-oxidation treatment contain broad diffraction bands at 2*θ* around 20–30° and 40–45° representative of graphitic crystallite with layered structure and the turbostratic graphite-like structure, respectively. These two bands are well defined in the reference carbon, while graphite shows mostly a sharp peak at 2*θ* around 20–30° [34]. The presence of these bands becomes flatter or even absent when the air oxidation is applied, indicating that this post-pyrolysis approach modifies the graphitic structure of biochars, introducing some oxygen-containing groups that weaken the structure.

The carbon content of biochars without the oxidation treatment is around 63–65%, determined by elemental analysis (Table 1), demonstrating a good carbonization degree of KBP biomass. In addition, a decrease in H and O contents occurs during the KBP pyrolysis due to dehydration reactions, the decomposition of oxygenated bonds, and the release of low molecular weight by-products [35]. However, when the air-oxidation treatment occurs at higher temperatures, biochar gradually decomposes by reacting with the air and, subsequently, it decreases the weight, leading to lower yields. Thus, the air oxidation treatment causes lower carbon content. Nevertheless, the 700C_3h_10air biochar sample does not show a severe carbon loss in contrast with the biochar samples oxidized during the same time, indicating a greater degree of aromatic carbon condensation during the pyrolysis, which is characterized with higher stability. This follows the H/C molar ratio, where a lower ratio value indicates a higher degree of aromaticity. Enhancing the air-oxidation time of biochar samples, the H/C ratio increases monotonously, which can be explained by the loss of a C element due to full oxidation to CO_2_ [36] or due to the opening of aromatic carbon ring structure, increasing the C–H linkages. Upon oxidation, the increase in ash content in biochar is observed due to the oxidation of mineral elements that originally belong to the biomass composition [37].

**Figure 3:**
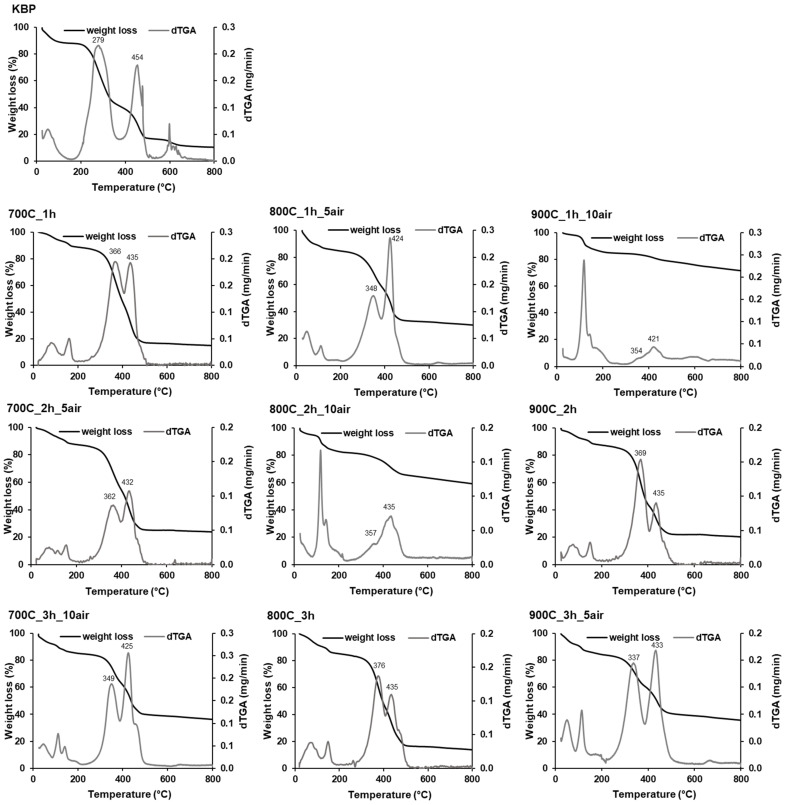
TGA/dTGA analysis of biomass (KBP) and biochars resulting from different pyrolysis conditions.

The rate of thermo-oxidative degradation of KBP, characterized by the dTGA curve (Figure 3), reflects four distinct weight-loss steps. The first step (<200 °C) corresponds to the mass loss caused by moisture evaporation. The second step, observed between 200 and 380 °C, is attributed to the oxidative fast devolatilization of hemicellulose and cellulose into volatiles and char. A third peak, observed with a maximum at 454 °C, is attributed to the combustion of produced char but also to the thermal degradation of lignin, which is a more complex structure compared to hemicellulose and cellulose [38]. The fourth thermal band, between 500 and 700 °C, corresponds to the degradation of minerals already present in KBP [39]. All the biochar TGA curves show a weight loss under 200 °C due to the removal of residual water. Above that, different degradations under thermal-oxidative atmosphere are observed considering the pyrolysis conditions. All biochars show degradation temperatures superior to the KBP, suggesting higher thermal stability. Biochar samples without the air-oxidative treatment (700C_1h, 900C_2h, and 800C_3h) evidenced a maximum weight loss rate in the interval of 330 to 390 °C, which occurs due to the degradation of thermally more unstable amorphous carbon compounds or of remaining organic compounds of biomass. In addition, another maximum rate with less intensity, around 410–430 °C, appears due to the recalcitrant aromatic moiety mass loss. These results indicate that these types of biochars are mainly made by alkyl organic material and aromatic domains. Conversely, the intensity of the two dTGA bands where the greatest degradation occurs is reversed when the air-oxidative treatment is applied on biochars, probably because some thermal unstable compounds were already degraded. The maximum decomposition temperature decreases for most of thermo-oxidized biochar samples, revealing their higher instability in consequence of the presence of O-containing groups and/or the condensation degree of the lower aromatic ring. Above 550 °C, all the biochar samples finished their decomposition, and the curves became stable. Thus, the obtained residue content corresponds to the ash content, which is higher with the increase in air-oxidation time after the pyrolysis treatment (Table 1). Concerning the DSC patterns, some biochar samples show an endothermic peak up to 200 °C, associated with the removal of water, and two exothermic peaks (one around 300–400 °C and another around 400–500 °C) due to the exothermic oxidation reactions occurred during the combustion of the biochar under an oxidizing atmosphere (Figure S3).

The highest S_BET_ was obtained for the 800C_1h_5air sample (54 m^2^/g), followed by 900C_3h_5air (23 m^2^/g) and 900C_2h (12 m^2^/g). The other biochar samples reported values less than 4 m^2^/g. To quantify the influence of the selected control factors (T, time, and air) on the S_BET_, the results were analyzed according to the Taguchi method. To do this, the mean signal-to-noise (S/N) ratio for each control factor was calculated and the data are plotted in Figure S4. By the analysis of the response graphs of the S/N ratio for “larger-is-better”, the greatest S/N variation indicates which control factor most influences the S_BET_ value. The degree of influence of each factor can be quantitatively ranked and the results indicate that the pyrolysis temperature is the most influential parameter, followed by the air-oxidation time and then by the residence time of pyrolysis (Table S1). Increasing the pyrolysis temperature generates a greater inner pressure in the biochar particles, which consequently allows the release of more volatiles, increasing S_BET_. This enhancement of textural properties of biochars with the increment of pyrolysis temperature was already reported [40]. Regarding the air-oxidative process, two competitive reactions can occur: the combustion of the biochar structure and the incorporation of oxygen atoms at the biochar surface [41]. Five minutes of air oxidation has a positive effect on the biochars’ surface properties, the S_BET_ increases probably due to an accelerated volatile release during the combustion, promoting the enhancement of free pores. However, increasing the air-oxidation time to 10 min, the combustion rate of biochar is higher than the diffusion of the compounds, negatively affecting the S_BET_ value. Moreover, a higher oxygen functionalization of biochar surfaces can also be responsible for decreasing the S_BET_ since to some extent these groups tend to fill the pores formed. Accurate control of the air-oxidative approach is a crucial factor to optimize the S_BET_ and to avoid uncontrolled combustion, as observed in a previous study [42]. Analysis of variance (ANOVA) (Table S2) was used to see the contribution of each factor and the results are consistent with the main effects of each factor, i.e., the pyrolysis temperature had the highest contribution (≈48%) followed by the time of air oxidation (≈29%), while the contribution percentage of residence time on the S_BET_ is too low (≈3%). Thereby, to achieve higher S_BET_ values compared to the commercial carbon, it would be interesting to explore higher pyrolysis temperatures associated with accurate control of air oxidative time process.

Raman spectra of biochar samples (Figure S5) were used to access their aromaticity since an increase in the I_D_/I_G_ ratio is an explicit indication of an increased structural order (graphitization) [43]. All biochar spectra exhibit two prominent overlapping bands lying around 1372 cm^−1^ and 1590 cm^−1^, representing the commonly called D and G bands of carbonaceous materials, respectively. When related to biochar structures, the first band is strictly connected to the C-C between the aromatic (benzene) rings and the presence of sixfold aromatic rings, while the second band is caused by the in-plane vibration of the sp^2^-hybridized graphitic carbon. The ratio of D band to G band (I_D_/I_G_) (Table 1) of all biochar samples is greater than one, which indicates that the carbon structure of biochar is predominantly composed of an amorphous carbon matrix with highly disordered graphitic-like crystallites. According to the response graphs of the S/N ratio for “larger is better” (Figure S6), the I_D_/I_G_ decreases when the temperature of pyrolysis and the air-oxidation time increase. The degree of influence of each factor was ranked and the results (Table S3) follow the percentage of contribution obtained by the ANOVA analysis (Table S4), where the most influent parameter is the air-oxidation time (58%), followed by pyrolysis temperature (22%) and then the residence time of pyrolysis (10%). Although a rise in the I_D_/I_G_ ratio has been observed with increasing pyrolysis temperature, as a consequence of the production of aromatic rings having six or more fused benzene rings, at temperatures above 700 °C a decrease in I_D_/I_G_ ratio has been demonstrated [44]. It has been described that when the temperature pyrolysis is greater than 700 °C, a higher aromatic ring condensation occurs where additional large aromatic ring systems having six or more fused benzene rings are produced. A change in a structural evolution from relatively small to large aromatic ring systems leads to a gradual graphitization of carbon structure over the pyrolysis temperature. This continued ring enlargement follows a decrease in the defect structure, decreasing the I_D_/I_G_ ratio [45]. On the other hand, the I_D_/I_G_ ratio of biochars drastically decreases when the air oxidation treatment is applied, indicating higher defects and disorders in the carbon structure. This severe and effective structural change in the biochar agrees with the conceptual “amorphization trajectory” [43]. During the air-oxidative process, the carbon loss that occurs opens the aromatic ring structure and destroys the condensation degree. This structural transition is under the higher H/C molar ratio, representing the decrease in graphitic crystallite size (sp^2^ carbon clusters) and the loss of polyaromatic carbon structure. In addition, the disordered graphitic structure increases also due to the surface oxygenation and O-containing functional groups on the aromatic ring structure. This functionalization promotes the presence of sp^3^ carbon bonds and a length disorder in the sp^2^ sites, which results in the FWHM (full width at half maximum) broadening of the G band (Table S5). The oxygen functionalization on the aromatic C by the air-oxidative process is also confirmed with the solid-state ^13^C NMR spectra, acquired by the CPMAS technique (Figure S7). The 800C_1h_5air biochar sample is predominantly aromatic since the spectrum is dominated by a single peak at 127 ppm, attributed to C- and H-substituted aromatic C. Other signals detected for the biochar were related to carbonyl groups at 140 ppm and carboxylic groups at 168 ppm, which can be assigned to O-substituted aromatic C, probably derived from the air-oxidation treatment. The 700C_3h_10air biochar sample presents the aromatic peak but the spectra also contain a strong and sharp peak in the carboxyl C group region at 168 ppm. For both samples, peaks around the alkyl region were not visible.

**Figure 4:**
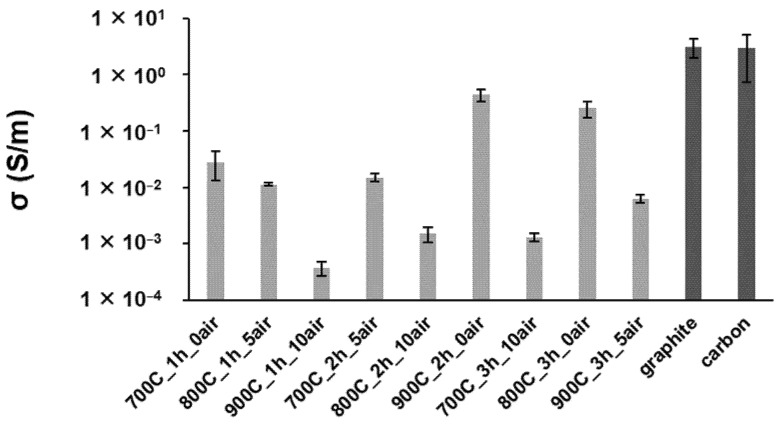
Electrical conductivity of biochar-sample pyrolysis with different time, temperature, and air-oxidation conditions (gray bars), with the graphite and carbon materials as reference (dark bars).

The effectiveness of biochars’ electrical conductivity measurement depends on many factors, including the packing density of powder, the crystalline structure, availability of electrons within the biochar structure, particle size, and surface elements (e.g., oxygen functional groups) [46]. In accordance with the S/N ratio analysis, where the criterion of “higher is better” was chosen (Figure S8), the most influential parameters are ranked according to the air-oxidation time, residence time, and temperature of pyrolysis (Table S6). Based on the ANOVA analysis (Table S7), the biggest percentage of contribution on the EC values is related to the air-oxidation time (87%) parameter, followed by the residence time of the pyrolysis factor (8%), and then the pyrolysis temperature (2%). Biochar samples without the air-oxidation treatment are those that showed the higher values of EC (Figure 4), which is related to the higher graphitization degree demonstrated above. In fact, the electrical conductivity of biochars is related to their graphitic-like structure [47]. The biochar that reached the highest EC value was the sample 900C_2h (0.45 ± 0.10 S/m), followed by 800C_3h (0.25 ± 0.08 S/m) and then by 700C_1h (0.03 ± 0.02 S/m). The best result obtained for EC is only one order of magnitude lower in comparison with the commercial graphite (3.13 ± 1.20 S/m) and carbon (2.94 ± 2.21 S/m). When looking at other reports and even comparing different feedstocks, the higher values obtained in this study are in the same order of magnitude as biochar produced from poultry litter (0.60 S/m) [48], but higher than the ones produced from miscanthus (9.5 × 10^−3^ S/m) [46]. With the increase in air-oxidation time, biochars reached lower values of EC because this post-pyrolysis treatment lowers the percentage of carbon content and increases the amorphization of the poorly graphitic structure, increasing the disordered carbon structure. According to ATR–FTIR, RAMAN, and ^13^C NMR data, the oxidative treatment increases the concentration of oxygen functional groups on the biochar surface and reduces the aromatic carbon structures. Therefore, the availability of electrons responsible for conducting electricity is lower, demonstrating an adverse impact on the EC. A study already demonstrated that EC of carbon black decreased when the size of the polyaromatic character is reduced and the presence of aliphatic groups on the surface of the carbon structure is enhanced [49]. Time and temperature of pyrolysis show that the *S*/*N* ratio slope is almost constant irrespective of levels, meaning that there is a low effect on EC values.

According to the above discussion, the produced biochars have different structural and functional characteristics that can be explored considering the design of an active filler to be incorporated into a biomaterial for food packaging. Considering this, two biochars were selected as an eco-friendly carbon platform to proceed with the synthesis of composites with zinc-oxide particles by an easy and green solvothermal methodology. The biochar samples chosen for the composite synthesis were 800h_1h_5air (C_SBET_) and 900h_2h (C_EC_) as per the experimental design of Taguchi (Table 2) due to the highest S_BET_ and EC values, respectively.

### 2.2. Characterization of ZnO-C Composites

The ZnO-C_SBET_ and ZnO-C_EC_ composites represent the zinc-oxide nanostructures using the C_SBET_ and C_EC_ biochar samples as a carbon platform, respectively. ZnO and ZnO-rGO particles were developed using the same methodology and characterized to compare their structure and functionality with the composites developed using the biochars.

**Figure 5:**
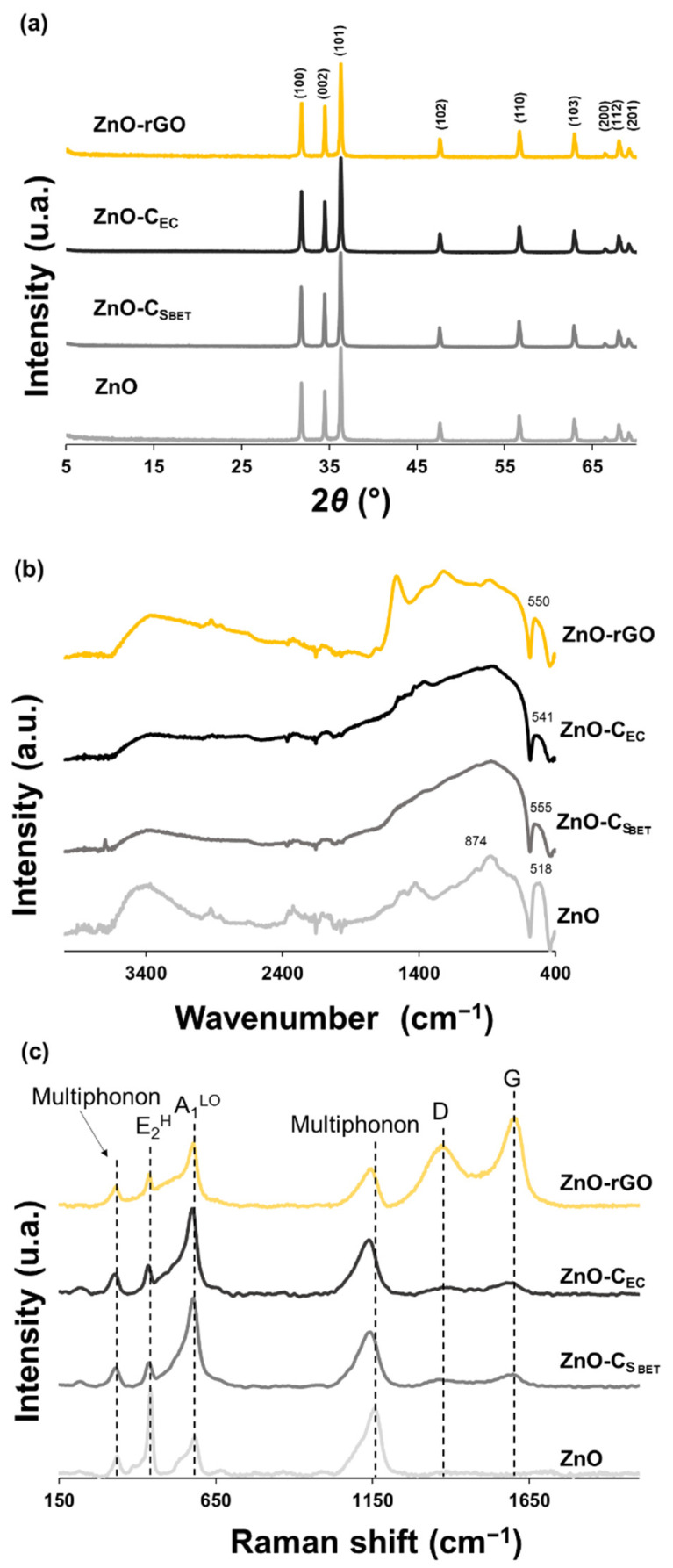
(**a**) XRD, (**b**) ATR–FTIR, and (**c**) Raman results of pristine ZnO and of ZnO-CS_BET_, ZnO-C_EC_, and ZnO-rGO composites.

Analysis of the structure and phase purity of biochar composites was performed with XRD to confirm the presence of ZnO particles on the biochars. Comparing the diffractograms of ZnO-C composites (Figure 5a) with the respective biochars (Figure S1) demonstrates that the ZnO particles were effectively synthesized using the biochars as a carbon template, since the displayed characteristic peaks are consistent with the hexagonal *wurtzite* structure of ZnO particles. The sharp and strong reflection of XRD peaks indicates that the ZnO particles were well crystallized using both biochar platforms, similar to when using the GO. The major diffraction peaks characterized at 2*θ* values around 31.8°, 34.5°, 36.3°, 47.6°, 56.6°, 62.9°, 66.4°, 67.9°, and 69.1° correspond to (100), (002), (101), (102), (110), (103), (200), (112), and (201) crystalline planes, respectively, and are consistent with the P*63*mc space group when compared to the representative peaks from Powder Diffraction Files database (JCPDS) card no. 36-1451. Additionally, the FWHM of the most intense crystallographic plane (101) was analyzed and it was concluded that the presence of carbon structures slightly increases this value, being more intense in the composites using C_EC_ biochar and GO as carbon structures (Table S8), suggesting that biochars can also affect the growth of ZnO structures. A shift to lower angles as well as a slight increase in the lattice parameters (Table S8) are observed in the ZnO-C_SBET_ composite when compared to the pristine ZnO. This suggests that accurate control of air oxidation to functionalize the surface biochar increases the coordination between the ZnO and the oxygen groups of the biochar surface, enabling different strains in the ZnO lattice. The X-ray fluorescence analyses clearly reveal that Zn was the most abundant element found in the samples, presenting an oxide-expressed content of around 95% and 94% for the pristine ZnO and ZnO-rGO samples and of 86% and 82% for the ZnO-C_SBET_ and ZnO-C_EC_ samples, respectively (Table S10). It should be noted that the lower weight percentage of ZnO on both biochar composites compared with ZnO-rGO results in higher content for other elements, such as CaO, P_2_O_5_, SiO_2_, MgO, and K_2_O. These metal oxides come from biochars used as support on the composites, corroborating the data obtained in their XRD patterns (Figure S1) and EDS analysis (Figure S2), which revealed the presence of diverse oxidative minerals in their structure due to the inherent elemental composition of KBP. Looking at the ATR–FTIR spectra (Figure 5b), the ZnO sample shows a peak at 879 cm^−1^ and an intense one around 518 cm^−1^ that are assigned to the metal-oxygen vibrational modes of ZnO particles. These bands are also present in both ZnO-C composites and concerning the band at around 518 cm^−1^, related to Zn–O vibrations, a shift to higher wavenumbers is observed when the ZnO is synthesized using biochar as carbon structure, indicating a coordination of ZnO with the functional groups of biochar, a result also observed for the ZnO-rGO composite [50]. These outcomes corroborate the analysis of XRD data, verifying a perturbation of the Zn–O–Zn network with the addition of carbon structures.

Figure 5c exhibits the Raman patterns of pristine ZnO particles and ZnO-C_SBET_, ZnO-C_EC_, and ZnO-rGO composites, which were performed to investigate the influence of carbon-based materials on the microscopic structure and vibrational properties of ZnO single crystals. The Raman spectrum of pristine ZnO particles displays four peaks located at 336, 441, 583, and 1155 cm^−1^ which are attributed to E_2_^H^-E_2_^L^ (multiphonon scattering), E_2_^H^, A_1_^LO^, and 2A_1_^LO^ and 2E_1_^LO^ (multiphonon), respectively [51]. The E_2_^H^ mode, the most intense sharp peak of pristine ZnO particles, confirms the formation of the hexagonal *wurtzite* structure of ZnO with high crystalline nature, since it represents the quality of O sub-lattice, while the A_1_^LO^ mode is commonly attributed to defect states (oxygen vacancy and/or zinc interstitials). In comparison to pristine ZnO particles, the composites show a shift to lower wavenumbers and a broadening of the ZnO-related peaks. Considering the E_2_^H^ mode, the inclusion of carbon-based materials decreases the peak intensity while increasing its broadening, suggesting the crystal damage of ZnO particles (Table S9). The rGO proved to be more injurious to ZnO crystallinity than biochars, probably because the larger size of the graphene sheets hinders the good growth of ZnO particles. In addition, with the inclusion of biochars, the ratio of E_2_^H^/A_1_^LO^ (Table S9) decreased more intensely than graphene oxide, suggesting that biochars induce the formation of native defects, namely zinc interstitials and oxygen vacancies. The lower ratio corresponds to the ZnO-C_SBET_ composite, which supports higher coordination of ZnO particles due to the presence of more oxygen-containing groups in the surface of biochar but also due to its higher surface area, which corroborates the data obtained with XRD analysis. In the spectra of composite samples there are two additional broad bands centered around 1363 cm^−1^ and 1598 cm^−1^, assigned to the characteristic D and G bands of amorphous carbon, respectively. These data confirm the presence of ZnO particles and carbon platforms in the composite samples.

Looking at the SEM micrographs of ZnO-C composites (Figure S9), both the selected biochars allowed the successful support of ZnO particles, forming ZnO-C composites. The ZnO particles in both biochar composites present a rod-shaped morphology with an appropriate distribution on the biochar surface, which is in accordance with the elemental mapping analysis of the C, Zn, and O elements. The interaction between the ZnO particles and the biochars as carbon support prevents the formation of some ZnO clusters which increases the specific surface area of the formed composites. As occurred with the ZnO-rGO composite (S_BET_ = 15 m^2^/g), both ZnO-C composites have a significantly larger S_BET_ (ZnO-C_SBET_ = 85 m^2^/g and ZnO-C_EC_ = 15 m^2^/g) than pristine ZnO particles (S_BET_ = 7 m^2^/g). In addition, the S_BET_ of biochar composites is higher than the biochar counterpart used as a platform, indicating less aggregation of ZnO particles due to its attachment on the biochar surface.

The optical properties were characterized with UV-vis diffusion reflectance spectroscopy (DRS) (Figure S10). Pure ZnO nanoparticles presents an absorption in the ultraviolet spectral region (Figure S10a) and a direct band gap of 3.19 eV calculated according to Tauc’s equation (Figure S10b), which is in agreement with the literature [52]. The incorporation of carbon materials into ZnO nanoparticles increases the absorbance in the visible light region and presents inferior band gap values, 3.11 eV for the ZnO-C_EC_ composite and the lowest value—3.08 eV—for the ZnO-C_SBET_ and ZnO-rGO composites. This means that in the case of ZnO composites the activation of electrons from the valance band to the conduction band is facilitated by the radiation source due to the formation of Zn-O-C chemical bonds in ZnO/carbon materials. Furthermore, it should be noted that the ZnO-C_SBET_ composite has a band gap value similar to ZnO-rGO, suggesting that biochar oxidation increases the interaction with ZnO, promoting a similar band-gap reduction when adding graphene sheets.

#### 2.2.1. Electrical Conductivity

**Figure 6:**
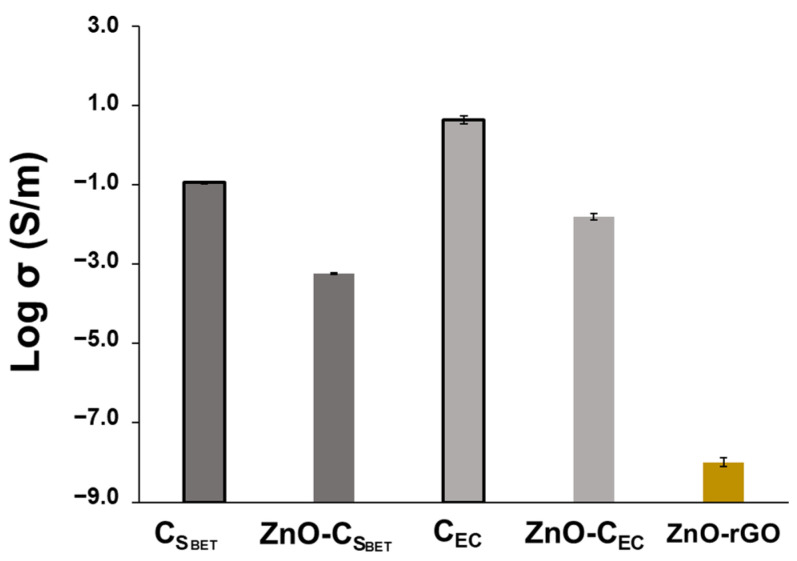
Electrical conductivity of biochar samples used as platform (C_SBET_ and C_EC_) and respective zinc composites (ZnO-C_SBET_ and ZnO-C_EC_). ZnO-rGO composite was used as a reference material (yellow bar).

Figure 6 compares the EC of ZnO-C_SBET_ and ZnO-C_EC_ biochar composites with the biochar counterpart and with the ZnO-rGO composite. Among the ZnO-C composites, the highest EC value is achieved by ZnO-C_EC_, following the conductivity tendency of the biochar counterpart. However, both ZnO-C composites demonstrate an EC value lower in one order of magnitude than the carbon biochar platform itself. The functionalization of biochars with ZnO particles reduces the EC due to the interruption of the conductivity network of the biochar carbon structure, with the ZnO acting as an insulator. This behavior has been observed for other composites based on carbon structures with metal oxide particles [53]. In contrast, both ZnO-C composites exhibit a much higher EC value, up to six orders of magnitude, than the ZnO-rGO composite. Considering that the measurement conditions were the same between samples, the ZnO-C composites showed enhanced efficiency on the electrical properties compared with the graphene composite (ZnO-rGO). This is a promising result to replace the graphene derivatives by biochars as a more environmentally friendly support for the metal-oxide-composite synthesis.

#### 2.2.2. Antimicrobial Activity

The antibacterial effect of biochar composites and ZnO-rGO was determined against two bacteria, *E. coli* ATCC25922 (Gram-negative) and *S. aureus* ATCC29213 (Gram-positive), after 24 h of contact with the bacterial suspension. Pristine ZnO particles were tested as a reference sample and the results obtained are shown in Figure S11. When compared to the bacterial suspension without any sample (control), *E. coli* ATCC25922 growth was not inhibited in any of the samples under study (log reduction was below one). In contrast, the growth of *S. aureus* ATCC29213 was completely inhibited with both ZnO-C composites and ZnO-rGO, suggesting a bactericidal effect on this bacterium. Additionally, all composites were demonstrated to have a more efficient antibacterial activity than the pristine ZnO and this could be explained probably by the higher S_BET_ of composites. Similar behavior was observed for Cu_2_O/biochar composites, which proved to be more effective in the antibacterial performance than Cu_2_O nanoparticles [54]. A high surface area provides active sites to facilitate the ZnO interaction and/or the diffusion of the Zn^2+^ elemental to interact with the bacteria cell walls, which consequently leads to greater antimicrobial activity [27]. In fact, the presence of biochars or graphene sheets avoids the serious aggregation problem of ZnO particles, making their surface more exposed and able to interact with the bacterium membrane. The exact bactericidal mechanism of the ZnO particles is not clearly known and is still controversial. Several mechanisms can be proposed, including the generation of reactive oxygen species (ROS), the release of Zn^2+^ ions, the direct contact of ZnO particles with cell walls or its internalization [55]. *S. aureus* ATCC29213 bacteria is sensitive to the presence of ZnO, ZnO-C_SBET_, ZnO-C_EC_, and ZnO-rGO, whereas no effect is observed against *E. coli* ATCC25922. This behavior was already reported in another study, which revealed that ZnO nanoparticles are more efficient in inhibiting the growth of *S. aureus* than those of *E. coli* [56]. This research raises the hypothesis that ZnO interacts with the positive charges of the Gram-positive bacteria cell membrane such as *S. aureus*, causing a decrease in its growth [56]. The similar performance of ZnO-rGO and of the two ZnO-C composites against the food-borne bacteria encourages the replacement of the graphene sheets by the biochar structures since it represents an eco-friendly and more biocompatible carbon support to produce the ZnO composites. It may be interesting to carry out a dose-dependent study to better understand whether the distinct surface area of the biochar composites shows a significant difference in the antibacterial efficacy.

## 3. Materials and Methods

### 3.1. Materials

Dried kidney-bean pods (KBPs) were collected from a local farmer in Oliveira de Azeméis, Portugal. The raw material was cut into small pieces, dried at 105 °C overnight to eliminate the remaining moisture, and ground. The zinc acetate, sodium hydroxide, and ethanol absolute were obtained from Sigma-Aldrich (St. Luis, MI, USA), LabChem Inc (Zelienople, PA, USA), and Carlo Erba (Barcelona, Spain), respectively. Graphite and carbon black (Vulcan XC72R) were purchased from Sigma-Aldrich (St. Louis, MI, USA) and Cabot Corp (Boston, MA, USA), respectively.

### 3.2. Preparation of Biochar

An experimental design based on the Taguchi orthogonal arrays was used to significantly reduce the number of trials and access the influence of determining factors on biochar features. The experiment was carried out using three operational pyrolysis parameters known as control factors with three levels: pyrolysis temperature (T)—700 °C, 800 °C, 900 °C; residence time (time)—1 h, 2 h, 3 h; and time of air oxidation (air)—0 min, 5 min, 10 min. This leads to nine pyrolysis experiments, summarized in Table 2. Dried and ground kidney-bean pods (KBPs) were the biomass pyrolyzed in a tubular furnace using alumina crucibles under nitrogen atmosphere. The air was purged from the reaction environment for 30 min and afterwards the furnace was turned on to raise the temperature following a pyrolysis program consisting of three steps: the first ramp of 5 °C/min with heating up to 150 °C, left at this temperature for 30 min to remove water; the second ramp with a heating rate of 5 °C/min up to 350 °C, maintaining at this temperature for 1 h to form the incipient conjugated carbon structure; and the third ramp at a rate of 5 °C/min reaching different target temperatures (700, 800, or 900 °C). Then, these temperatures were sustained for a holding time of 1, 2, or 3 h to produce polyaromatic carbon structures. A thermal oxidation process was performed under air flow for 5 or 10 min, as a post-pyrolysis treatment to modify the biochar samples. Subsequently, all pyrolyzed samples were cooled down to room temperature under nitrogen flow.

### 3.3. Analysis of Taguchi Design

The results of S_BET_, I_D_/I_G_ ratio, and EC parameters were selected as output responses of the biochar’s properties with interest towards the functionalization with ZnO particles, having the goal of synthesizing an active and electrically conductive composite filler. The Taguchi method employs a generic signal-to-noise (S/N) ratio, which represents the ratio of mean response (signal) to the standard deviation (noise), as a quantitative measure to determine the relative importance of factors on the selected output responses [57]. The selection principles of the S/N ratio, which are divided into three categories (“larger-is-better”, “nominal-is-better”, and “smaller-is-better”), depends on the goal of the study. The experiments were designed to maximize the S_BET_ and EC, so the “larger-is-better” approach was adopted, in which the S/N ratio is calculated from Equation (1):

(1) (SN)larger−is−better=−10 log10 [1n ∑i=1n(1Yi2)],

where *S*/*N* indicates the signal-to-noise statistic used in the Taguchi parameter analysis, *n* is the number of repetitions performed for an experiment, and *Y_i_* is the value of the output response (i.e., S_BET_ or EC) of each experiment [57]. In the case of the I_D_/I_G_ ratio, which can indicate biochar graphitization degree, the “larger-is-better” approach was also selected considering the few defects in the biochar structure. Analysis of variance (ANOVA) of S/N ratios can be used to study the most significant factors of pyrolysis conditions affecting the output responses. The Minitab software was used to analyze the results obtained using the Taguchi method.

### 3.4. Preparation of ZnO-Biochar

The biochars with the highest surface area (800C_1h_5air now denoted as C_SBET_) and EC (900C_2h now denoted as C_EC_) were chosen to be used as a carbon template to synthesize ZnO particles under a solvothermal methodology. Briefly, a solution of zinc acetate (Zn(CH_3_COO)_2_) at 0.1 M and a solution of sodium hydroxide (NaOH) at 2 M were prepared in ethanol. For 10 mL of zinc precursor solution, 1.6 mL of biochar aqueous suspension (12.5 mg/mL), which was previously sonicated for 5 min, was added dropwise. After 30 min of stirring, 20 mL of NaOH solution was added dropwise under vigorous stirring and the reaction took 1 h. The solution was transferred to a Teflon-lined stainless-steel autoclave (60 mL) and heated in an oven at 150 °C for 24 h to produce ZnO structures impregnated in biochar. The produced ZnO-C composites were collected, washed with water and ethanol several times to neutralize the pH, and then dried at 60 °C to be further characterized. Synthesis of pure ZnO was also performed using the same procedure replacing the biochar suspension with 1.6 mL of distilled water. A sample with graphene oxide (GO) instead of biochar was also collected for comparative studies.

### 3.5. Characterization of Biomass, Biochars, and ZnO-C Composites

Fourier Transform Infrared (FTIR) spectra of samples were acquired using a Golden Gate single reflection diamond attenuated total reflectance (ATR) system in a Bruker IFS-55 spectrometer. Spectra were recorded at the absorbance mode from 4000 to 400 cm^−1^ wavenumber (mid-infrared region) with a resolution of 4 cm^−1^. Five replicates (64 co-added scans) were collected for each sample.

Powder X-ray diffraction (XRD) was performed on a Panalytical Empyrean X-ray diffractometer with Cu-Ka radiation ((λ = 1.54178 Å). The diffractograms were recorded in a reflection mode with a scanning angle ranging from 5 to 70° 2*θ*. For phase identification, an integrated database of Powder Diffraction Files (JCPDS) from the International Centre of Data Diffraction (ICDD) was employed.

Elemental analyses of samples were performed with a TruSpec 630-200-200 CHNS Analyzer, using a sample amount between 1 and 2 mg. The temperature of the combustion furnace and temperature afterburner was 1075 °C and 850 °C, respectively. The detection methods applied were carbon infrared absorption, hydrogen infrared absorption, nitrogen thermal conductivity. The oxygen (%) content was determined using the conventional difference method, where O (%) = 100–H (%)–C (%)–N (%)–ash (%).

Thermogravimetric analysis (TGA) and Differential Scanning Calorimetry (DSC) were carried out with a Hitachi STA300 instrument (Hitachi, Tokyo, Japan) at a heating rate of 10 °C min^−1^ from room temperature to 800 °C, under air atmosphere. The final inorganic residue mass obtained by TGA was used to quantify the ash content of biochar samples.

For the ash determination of kidney-bean pods, this biomass was dried at 105 °C for 16 h in ceramic crucibles. After cooling to room temperature on a desiccator, the crucibles were weighed to determine moisture content by gravimetry. Then, the dried biomass was incinerated in a muffle furnace at 575 °C for 6 h, cooled to room temperature, and weighed. This measurement was performed in triplicate and the following value was obtained: 7.65% ± 0.08%.

The ^13^C NMR spectra were recorded on a Bruker Avance III 400 spectrometer operating at 9.4 T at 100.62 MHz. ^13^C cross-polarization (CP) magic-angle spinning (MAS) NMR spectra were obtained with 4 µs ^1^H 90° pulse, contact time (CT) of 1 ms, a spinning rate (νR) of 12 kHz, and a recycle delay (RD) of 4 s.

The BET (Brunauer–Emmett–Teller) method was used for the calculation of the specific surface area (S_BET_). The −196 °C nitrogen adsorption/desorption isotherms were acquired using a Micromeritics^®^–Gemini 2380 V2.00 and the samples degassed at 120 °C overnight.

Raman spectroscopy was carried out using the Jobin Yvon T64000 instrument equipped with a laser operating at 441 nm as an excitation source wavelength laser. At least three replicas were performed for each sample.

X-ray fluorescence spectroscopy was carried out with a Panalytical Axios spectrometer PW4400/40 X-ray (Marvel Panalytical, Almelo, The Netherlands), equipped with Rh tube under argon/methane. The analysis of mineral composition was obtained using the Omnian software.

Electrical conductivity was measured by placing the sample powder in a home-made resistive setup consisting of an insulating tube with 0.7 cm diameter where the sample was in contact with and pressed between two aluminum pistons. The resistance of the samples was measured at room temperature with direct current (DC) using a programmable power supply IPS603 (ISO-Tech) and a Multimeter 34401A (HP). The value of electrical conductivity (S/m) for each film was then calculated following Equations (2) and (3).

(2) $$R=\frac{V}{I}$$

(3) $$\sigma =\frac{L}{R\times A}$$

where *R* is the resistance, *V* means tension, *I* is current, σ is electrical conductivity, *L* and *A* are the powder height and the cross-sectional area for each specimen in the tube.

Scanning electron microscopy (SEM) was employed to observe the morphology of ZnO-C composite samples using a field-emission gun SEM Hitachi SU70 microscope operated at 15 kV. The elemental compound was investigated using energy dispersive spectroscopy (EDS, Bruker Quantax 400). Samples were deposited on a sample holder and coated with evaporated carbon.

The antibacterial activity was examined concerning the ability to inhibit the growth of two food-borne pathogenic bacteria, *Escherichia coli* (ATCC25922) (Gram-negative) and *Staphylococcus aureus* (ATCC29213) (Gram-positive). Two to three pure colonies of each bacterium were aseptically inoculated in 20 mL of TSB broth and incubated at 37 °C for 16 h. The inoculum was aseptically transferred to 2.5 mL of TSB broth to obtain an initial concentration of bacteria ~10^3^ CFU mL^−1^. Then, 15 mg of each sample was added to the previously inoculated broth and incubated at 37 °C for 24 h with shaking (180 rpm). The number of viable bacteria was estimated with single plate-serial dilution spotting (SP-SDS). For concentrated samples, an anchoring at 0.1 OD was carried out and serial dilutions were performed (10^1^–10^5^). A 20 µL amount of the three most diluted solutions was applied as 9–13 micro-drops in a respective sector over a TSA (Tryptic Soy Agar) medium and incubated at 37 °C for 16–24 h. In each sector, the colonies were counted considering the acceptable number of colonies in each dilution (6–60 per sector) [58]. Control samples consisted in the culture broth without any sample to test. The assay was performed in triplicate for each sample.

## 4. Conclusions

The pyrolysis of kidney-bean pods by applying an experimental design demonstrates that S_BET_ of biochars was enhanced when the pyrolysis temperature increased along with a short time of air oxidation. On other hand, increasing the time of air oxidation decreased the graphitization degree and electrical conductivity of biochars. Besides the identical efficacy against the *S. aureus* microorganism, the prepared ZnO-C_SBET_ and ZnO-C_EC_ composites effectively demonstrated a higher S_BET_ and EC, respectively, when compared to the ZnO-rGO composite. The results herein presented show the potential of replacing the traditional carbon materials by biochars as an efficient and sustainable resource, aiming at the production of an active and electrically conductive filler for reinforcing bioplastic materials.

## Figures and Tables

**Table 1:** Yield (%), S_BET_ (m^2^/g), I_D_/I_G_ ratio, and content (%) of C, H, N, O, and ash of the biochars, commercial graphite, and carbon.

Sample	Yield (%)	S_BET_ (m^2^/g)	I_D_/I_G_	C (%)	H (%)	N (%)	O (%)	H/C	Ash (%)
KBP	-	2	-	38.08	5.14	0.54	48.59	1.61	7.7
700C_1h	71	2	3.17	64.76	1.45	0.88	17.95	0.27	15.0
800C_1h_5air	55	54	2.36	55.85	1.37	1.01	11.75	0.29	30.0
900C_1h_10air	22	4	1.33	6.15	1.72	0.00	20.53	3.34	71.6
700C_2h_5air	54	2	2.84	57.77	1.46	0.96	16.00	0.30	23.8
800C_2h_10air	20	4	1.75	18.00	1.51	0.45	20.88	1.00	59.2
900C_2h	65	12	3.28	65.43	1.10	0.91	15.09	0.20	17.5
700C_3h_10air	38	2	2.77	49.43	1.28	1.10	11.92	0.31	36.3
800C_3h	68	4	3.12	63.45	1.47	0.86	18.38	0.28	15.8
900C_3h_5air	38	23	2.19	42.69	1.56	0.91	19.17	0.44	35.7
Graphite	-	8	0.04	95.57	n.d.	n.d.	-	-	-
Carbon	-	246	2.05	95.64	n.d.	n.d.	-	-	-

Measurements performed in triplicate, but standard deviations are not presented in the table for simplification proposes. n.d.—not detected.

**Table 2:** Experimental design.

	Process Parameters			
Run	T (°C)	Time (h)	Air (min)	Sample Name
1	700	1	0	700C_1h
2	800	1	5	800C_1h_5air
3	900	1	10	900C_1h_10air
4	700	2	5	700C_2h_5air
5	800	2	10	800C_2h_10air
6	900	2	0	900C_2h
7	700	3	10	700C_3h_10air
8	800	3	0	800C_3h
9	900	3	5	900C_3h_air

## Data Availability

Not applicable.

## Supplementary Materials

The following supporting information can be downloaded at: https://www.mdpi.com/article/10.3390/ijms23148022/s1.
