# Supplementary material for: Electrically Conductive and Antimicrobial Agro-Food Waste Biochar Functionalized with Zinc Oxide Particles
Source: Int J Mol Sci. 2022 Jul 21;23(14):8022. doi: 10.3390/ijms23148022 (PMC9319753; doi:10.3390/ijms23148022)
Supplement: Supplementary file 1 [file ijms-23-08022-s001.zip › ijms-1760075-supplementary.pdf]

Supplementary Information

Electrically conductive and antimicrobial agro-food waste biochar functionalized with zinc oxide particles

Zélia Alves[1,2], Nuno M. Ferreira[3], Gonçalo Figueiredo[4], Sónia Mendo[4], Cláudia Nunes[1,*], Paula Ferreira[1,*]

[1] CICECO – Aveiro Institute of Materials, Department of Materials and Ceramic Engineering, University of Aveiro, 3810-193 Aveiro, Portugal

[2] CICECO – Aveiro Institute of Materials, Department of Chemistry, University of Aveiro, 3810-193 Aveiro, Portugal

[3] i3N, Department of Physics, University of Aveiro, 3810-193 Aveiro, Portugal

[4] CESAM, Department of Biology, University of Aveiro, 3810-193 Aveiro, Portugal

**Corresponding authors:*

Cláudia Nunes, claudianunes@ua.pt

Paula Ferreira, pcferreira@ua.pt;

1. Results and Discussion

1.1. Characterization of biochars

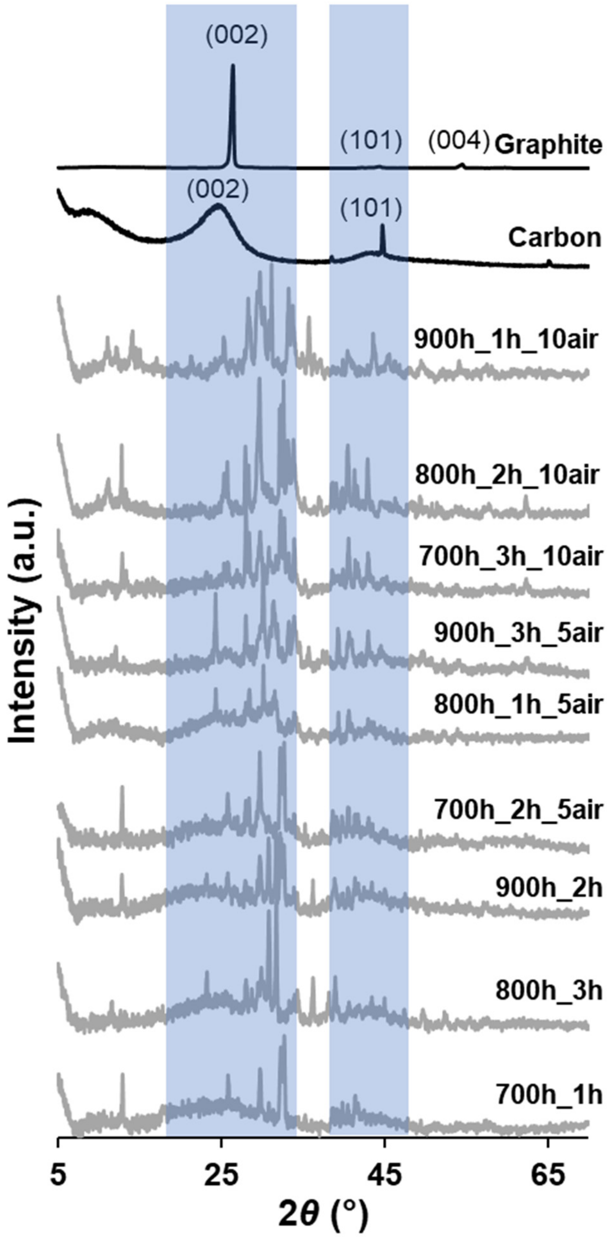

Figure S1 – XRD diffractograms of biochars resultant of kidney bean pods pyrolysis using different time, temperature, and air oxidation conditions. Diffractograms of carbon and graphite are displayed as a reference.

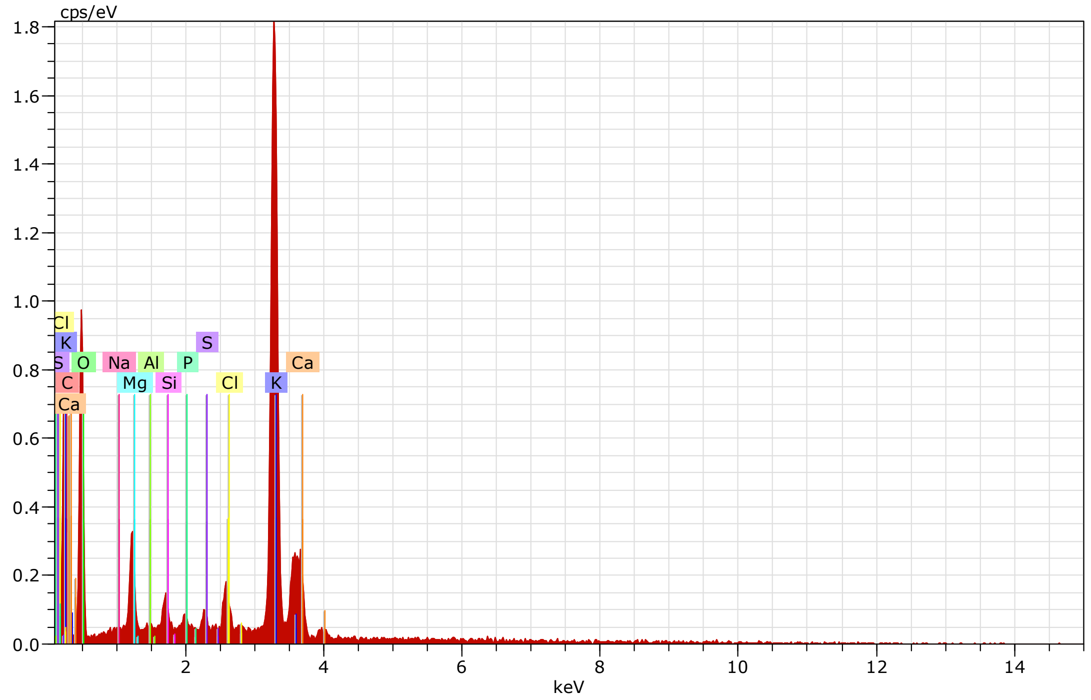

Figure S2 – EDS analysis of 800C_2h_10air biochar sample.

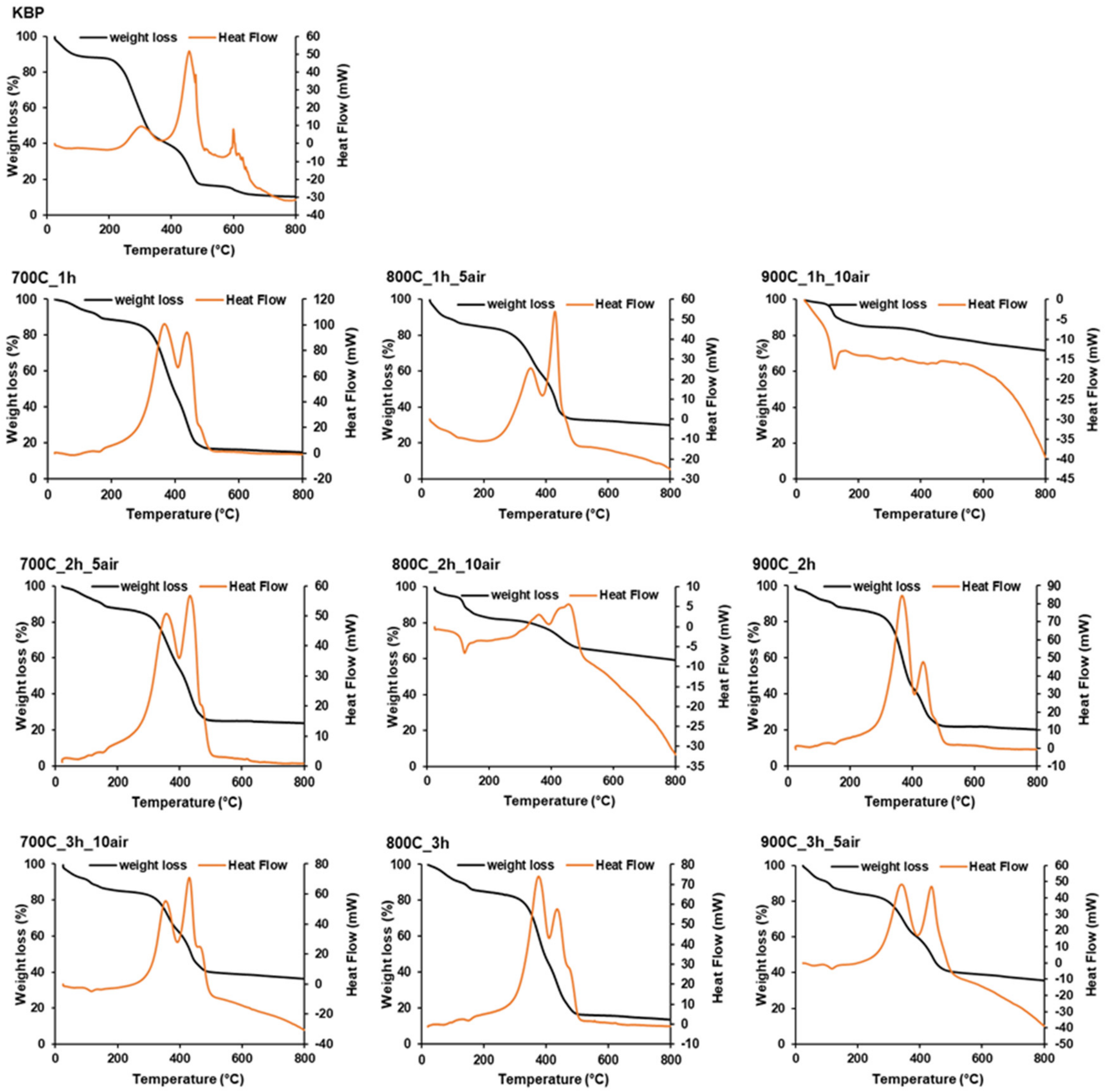

Figure S3 – TGA-DSC analysis of biomass (KBP) and biochars resulted from different pyrolysis conditions.

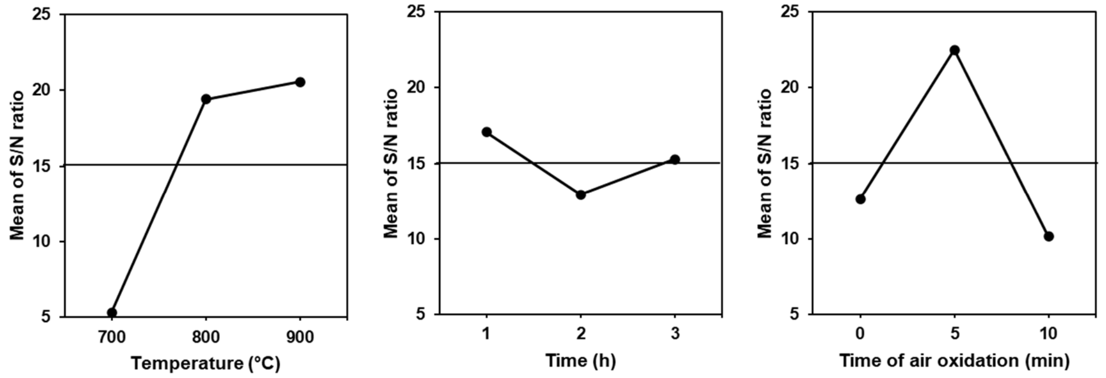

Figure S4 – S/N ratio graph (larger is better) of the control factors on the S_{BET} response.

Table S1 – Response Table for Signal to Noise Ratios - Larger is better

Level	Temperature	Time	Time of air oxidation
**1**	5.318	17.069	12.632
**2**	19.411	12.94	22.492
**3**	20.557	15.278	10.162
**Delta**	15.239	4.129	12.33
**Rank**	1	3	2

Table S2 – Analysis of Variance for S/N ratios related with S_{BET}.

Source	DF	Seq SS	Adj SS	Adj MS	F	P	Contribution (%)
**Temperature**	2	432.15	432.15	216.07	2.52	0.284	48.85
**Time**	2	25.72	25.72	12.86	0.15	0.87	2.91
**Time of air oxidation**	2	255.36	255.36	127.68	1.49	0.402	28.86
**Residual Error**	2	171.46	171.46	85.73			19.38
**Total**	8	884.69					

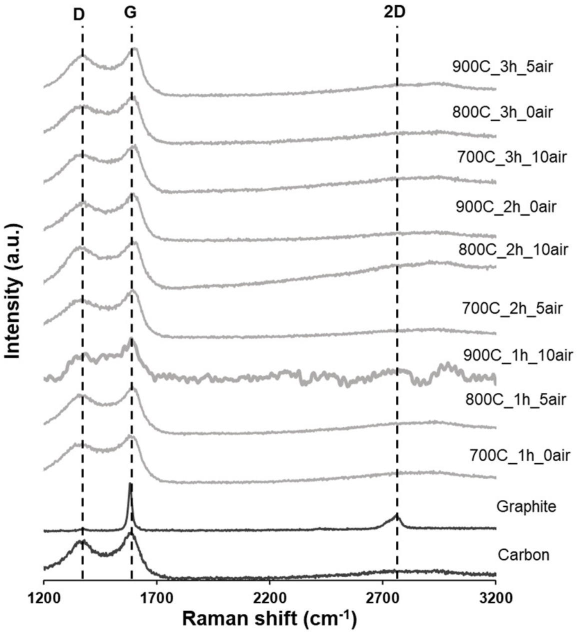

Figure S5 – Raman spectra of biochars resulted from pyrolysis of kidney bean pods in different conditions. The spectra of carbon and graphite are displayed as a reference.

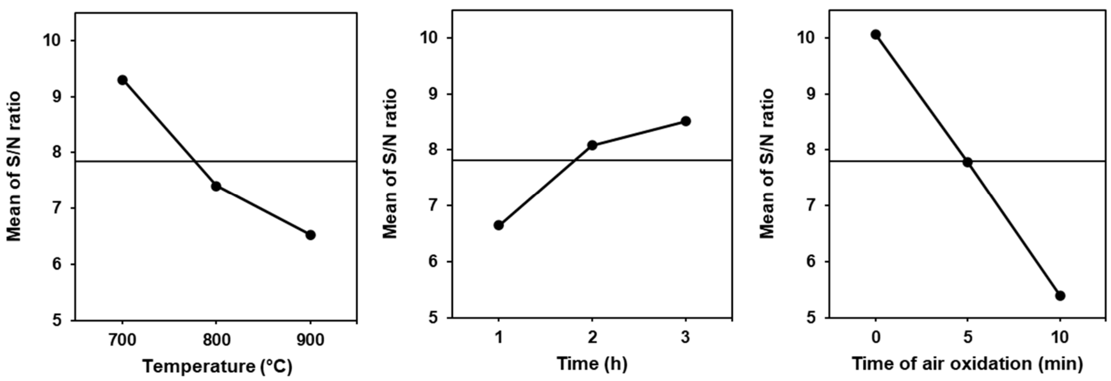

Figure S6 – S/N ratio graph (larger is better) of the control factors on the I_D/I_G response.

Table S3 – Response Table for Signal to Noise Ratios - Larger is better of the control factors on the I_D/I_G response.

Level	Temperature	Time	Time of air oxidation
**1**	9.304	6.648	10.069
**2**	7.406	8.082	7.776
**3**	6.532	8.512	5.398
**Delta**	2.772	1.865	4.671
**Rank**	2	3	1

Table S4 – Analysis of Variance for S/N ratios related with I_D/I_G response.

Source	DF	Seq SS	Adj SS	Adj MS	F	P	contribution (%)
**Temperature**	2	12.051	12.051	6.026	2.14	0.319	21.47
**Time**	2	5.72	5.72	2.86	1.01	0.497	10.19
**Time of air oxidation**	2	32.726	32.726	16.363	5.8	0.147	58.29
**Residual Error**	2	5.644	5.644	2.822			10.05
**Total**	8	56.142					

Table S5 – Raman data (FWHM) relative to D and G band of biochars, and carbon and graphite used as a reference.

	FWHM (cm^{-1})	
	D	G
**700C_1h_0air**	307.265	105.1851
**800C_1h_5air**	263.7241	109.0252
**900C_1h_10air**	187.38	113.5524
**700C_2h_5air**	323.429	93.29312
**800C_2h_10air**	217.7947	119.4004
**900C_2h_0air**	305.8885	94.81156
**700C_3h_10air**	277.4096	102.6228
**800C_3h_0air**	296.4806	99.08171
**900C_3h_5air**	245.9497	103.1201
**Graphite**	28.53378	22.3247
**Carbon**	248.3008	101.9279

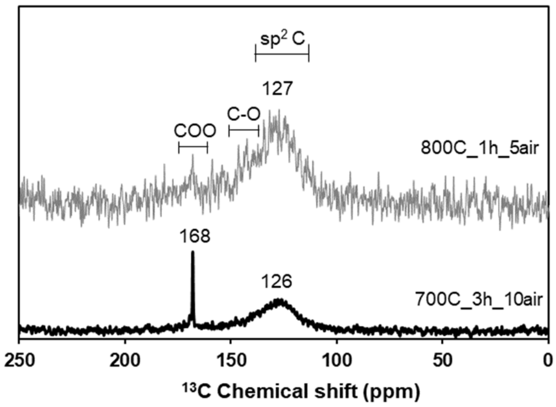

Figure S7 – ^{13}C CP-MAS NMR spectra of 800C_1h_5air and 700C_3h_10air biochar samples.

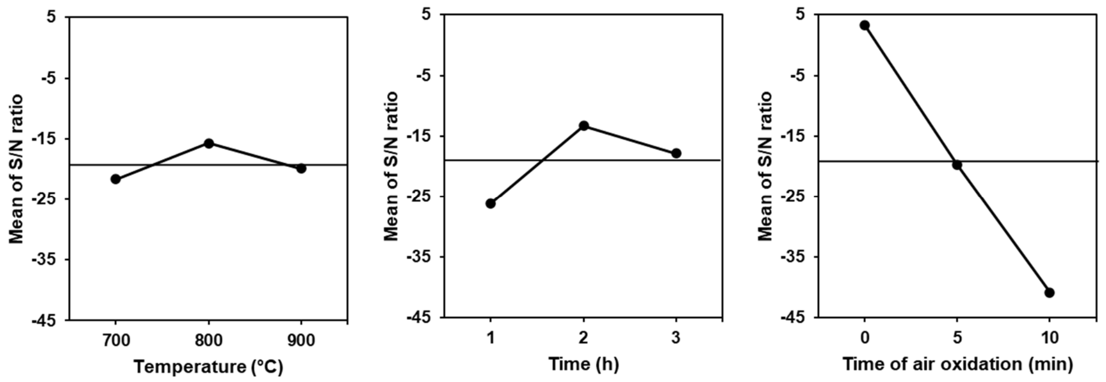

Figure S8 – S/N ratio graph (larger is better) of the control factors on the electrical conductivity response.

Table S6 – Response Table for Signal to Noise Ratios - Larger is better of the control factors on the electrical conductivity response.

Level	Temperature	Time	Time of air oxidation
**1**	-21.666	-26.133	3.292
**2**	-15.726	-13.325	-19.762
**3**	-19.891	-17.825	-40.813
**Delta**	5.939	12.809	44.105
**Rank**	3	2	1

Table S7 – Analysis of Variance for S/N ratios related with electrical conductivity response.

Source	DF	Seq SS	Adj SS	Adj MS	F	P	Contribution (%)
**Temperature**	2	55.77	55.77	27.89	0.44	0.695	1.66%
**Time**	2	253.34	253.34	126.67	2	0.334	7.55%
**Time of air oxidation**	2	2919.83	2919.83	1459.92	23.01	0.042	87.01%
**Residual Error**	2	126.91	126.91	63.45			3.78%
**Total**	8	3355.85					

1.2. Characterization of ZnO-C composites

Table S8 – FWHM of (101) plane, lattice parameters and S_{BET} present in the XRD diffractograms of pristine ZnO and ZnO-CS_{BET}, ZnO-C_{EC}, and ZnO-rGO composites.

	(101) FWHM (°)	Lattice parameters (Å)		S_{BET} (m^2/g)
		a=b	*c*	
**ZnO**	0.22583	3.243	5.193	7
**ZnO-CS_{BET}**	0.23767	3.246	5.200	85
**ZnO-C_{EC}**	0.256657	3.243	5.196	15
**ZnO-rGO**	0.25299	3.243	5.193	15

Table S9 – Raman data of pristine ZnO and ZnO-CS_{BET}, ZnO-C_{EC}, and ZnO-rGO composites samples related with the modes of ZnO and rGO.

	E_2^H FWHM	E_2^H/A_1^{LO}	FWHM		I_D/I_G
			D	G	
**ZnO**	15.322	2.174	-	-	-
**ZnO-CS_{BET}**	16.974	0.140	214	103	1.36
**ZnO-C_{EC}**	18.297	0.168	134	103	0.78
**ZnO-rGO**	23.027	0.525	165	88	1.36

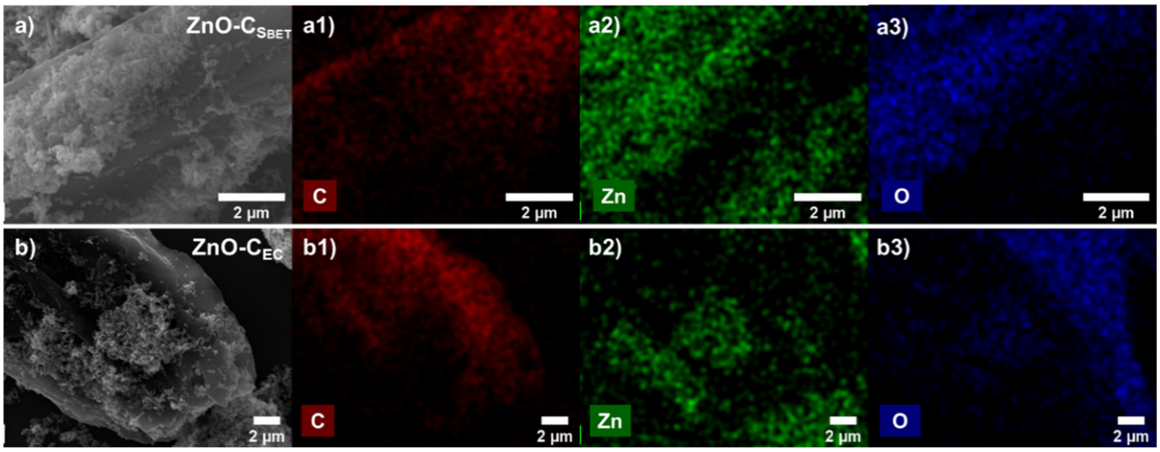

Figure S9 – SEM images of (a) ZnO-CS_{BET} and (b) ZnO-C_{EC} composites and corresponding EDS elemental maps of C, Zn, and O elements.

Table S10 – X-ray fluorescence spectroscopy data of pristine ZnO and zinc oxide composites, namely ZnO-C_{SBET}, ZnO-C_{EC}, and ZnO-rGO.

	Samples			
	**ZnO**	**ZnO-C_{SBET}**	**ZnO-C_{EC}**	**ZnO-rGO**
**Na_2O** (%)	-	-	-	0.55
**MgO** (%)	-	0.42	1.00	0.29
**Al_2O_3** (%)	0.05	-	0.11	-
**SiO_2** (%)	0.35	0.69	1.25	0.42
**P_2O_5** (%)	2.98	5.70	6.99	2.34
**SO_3** (%)	-	0.18	0.51	0.14
**Cl** (%)	-	0.3	0.59	0.20
**K_2O** (%)	-	0.1	0.14	0.07
**CaO** (%)	1.13	5.23	6.36	1.63
**TiO_2** (%)	-	-	0.36	-
**Fe_2O_3** (%)	0.14	0.33	0.45	0.08
**NiO** (%)	0.03	0.05	0.10	0.04
**CuO** (%)	-	-	0.09	0.03
**SrO** (%)	-	0.12	-	-
**PdO** (%)	-	0.49	-	-
**ZnO** (%)	95.34	86.39	82.07	94.21

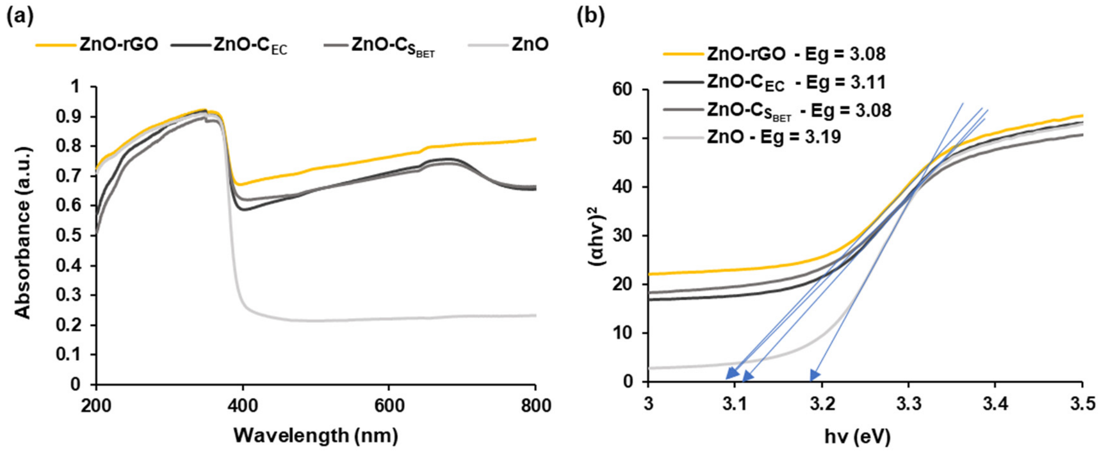

Figure S10 – (a) UV-vis diffuse reflectance (DRS) and (b) Tauc plots of ZnO, ZnO-C_{SBET}, ZnO-C_{EC}, and ZnO-rGO composites samples.

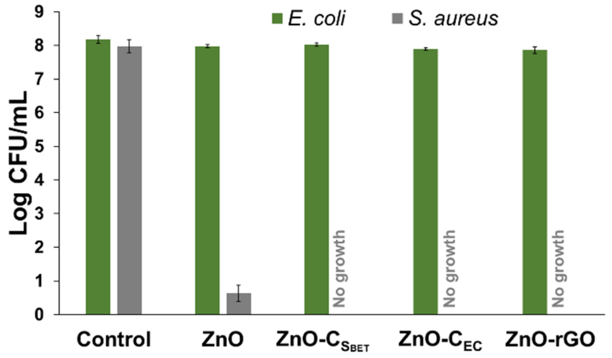

Figure S11 – Antibacterial activity against *E. coli* ATCC25922 and *S. aureus* ATCC29213 of pristine ZnO and the zinc oxide composites, namely ZnO-C_{SBET}, ZnO-C_{EC}, and ZnO-rGO. The control consisted only on each bacterial strain growing in the absence of any composites (Control).
